# Isolationand Identification of Antagonistic Bacteria Against *Sporisorium scitamineum* and Their Biocontrol Effect on Sugarcane Smut

**DOI:** 10.3390/plants15132091

**Published:** 2026-07-05

**Authors:** Wen-Shuo Yuan, Yong-Jia Li, Jia-Xin Li, Xiao-Hui Huang, Wan-Kuan Shen

**Affiliations:** 1 College of Agriculture, South China Agricultural University, Guangzhou 510642, China; wsyuan155@163.com (W.-S.Y.); 18229329252@163.com (Y.-J.L.); leegahun@126.com (J.-X.L.); 15718110884@163.com (X.-H.H.); 2 Sugarcane Research Laboratory, South China Agricultural University, Guangzhou 510642, China

**Keywords:** sugarcane smut disease, rhizosphere microorganisms, fluorescent staining, biological control, antimicrobial spectrum

## Abstract

Sugarcane smut is a fungal disease caused by *Sporisorium scitamineum*. To explore its biological control strategies, this study collected rhizosphere soil of sugarcane, isolated and identified biocontrol bacteria from it, and conducted multi-level control efficacy evaluations. The results showed that five bacterial strains with effective antagonistic activity against the sexual mating and teliospore germination of *S. scitamineum* were isolated and identified: 2143-2 (*Pseudomonas baetica*), 2143-4 (*Bacillus subtilis*), 2143-6 (*Burkholderia diffusa*), Y8-2 (*Pseudomonas reinekei*), and Y8-3 (*Bacillus amyloliquefaciens*). The results of the pot inoculation experiments showed that all five strains could prolong the incubation period of sugarcane smut and significantly reduce the disease incidence, demonstrating marked control effects, with strains 2143-4 and Y8-2 being the most effective. The results of the field inoculation experiments and natural field infection experiments indicated that strain Y8-2 exhibited the best biocontrol efficacy against sugarcane smut, with control efficacies of 68.40% and 73.99% in the field inoculation experiments, and 65.73% under natural field infection conditions. In addition, the biocontrol strains could improve the physiological stress-tolerance characteristics of sugarcane plants, which was conducive to enhancing the resistance of sugarcane plants to sugarcane smut.

## 1. Introduction

Sugarcane (*Saccharum* spp. hybrids), the world’s most vital crop for sugar and energy production, is also widely used in manufacturing sucrose, ethanol, biofuel, and fiber-based products [1]. Sugarcane is mainly distributed in tropical and subtropical regions, and the main sugarcane-growing areas in China include south-central Guangxi, southwestern Yunnan, western Guangdong, and northern Hainan. Guangxi is the largest sugarcane production area in China, accounting for 60% of production in China [2]. In addition to temperature and humidity, pests and diseases are among the most critical factors affecting yield during sugarcane growth. Among these, sugarcane smut, caused by the fungus *Sporisorium scitamineum*, is a globally significant sugarcane disease.

*Sporisorium scitamineum* belongs to the Basidiomycetes, and sugarcane smut caused by this fungus seriously affects sugarcane production [3]. The disease was first discovered in Natal, South Africa, in 1877 and remained confined to sugarcane-producing regions in the Eastern Hemisphere until 1940, when it spread to the Western Hemisphere [4]. This disease was reported in Guangzhou, China, in 1932, followed by reports in Guangxi, Yunnan, Fujian, and Taiwan [4,5]. Sugarcane smut is caused by teliospore infection, which do not germinate under low temperatures but can survive for over 6 months in hot and dry environments [6]. Smut symptoms are difficult to observe in the early stages of infection. Infected sugarcane plants have slender leaves, thin stalks, and excessive tillering. After 3–4 months of growth, a black whip-like structure, ranging from a few centimeters to several tens of centimeters in length, emerges at the apex and is covered with a large number of teliospores [7]. The smut whip reaches its maximum length at 6–7 months after pathogen infection [8]. Sugarcane smut reduces sugarcane yield and the sucrose content, seriously affecting yield, quality, and ratoon performance, and the impact on yield increases with the increase in the number of ratoon years [9].

The rhizosphere primarily refers to the narrow soil zone surrounding plant roots that is directly influenced by root activity, exhibiting distinct properties compared to bulk soil. Microorganisms colonizing this region are called rhizosphere microorganisms [10]. As a distinct microenvironment, the sugarcane rhizosphere harbors a more abundant and biologically active microbial community than the bulk soil [11]. Rhizosphere bacteria are one of the main components of rhizosphere microorganisms, participating in or affecting most biochemical reactions in soil, such as secreting plant hormones and activating soil nutrients [12,13]. Although there are few rhizosphere fungi, they are an important part of rhizosphere microbial diversity [14].

Biological control is a plant disease management approach that utilizes living organisms (animals, plants, or microorganisms) or their metabolites to suppress phytopathogens [15]. Compared to conventional chemical control, biological control reduces the dependence on chemical pesticides without leaving harmful residues in the environment. This approach mitigates ecological pollution, preserves biodiversity, and has advantages, such as safety, efficacy, and environmental sustainability. As a green and sustainable management strategy, it has significant potential for widespread application [16]. Biological control for sugarcane smut has attracted increasing attention. Rhizosphere bacteria are mainly used to antagonize *S*. *scitamineum* and inhibit sexual mating and mycelial growth, thus preventing and controlling sugarcane smut. Bacterial strain ST4 isolated from the sugarcane rhizosphere by Liu et al. can produce antagonistic chemicals when inoculated with exogenous glucose, inhibiting the sexual mating of *S*. *scitamineum*. Lin et al. constructed the ST4E strain to utilize the host’s sucrose, thus improving the inhibition of *S*. *scitamineum* [17,18]. *Pseudomonas aeruginosa* B18, an endophytic strain isolated from sugarcane roots, enhances host resistance against *S*. *scitamineum* and stably colonizes the sugarcane rhizosphere. Furthermore, this bacterial strain promotes sugarcane growth by modulating phytohormone production [19]. Bacteria antagonistic to *S*. *scitamineum* are widely distributed. Jiang et al. [20] isolated 19 bacterial strains exhibiting inhibitory activity against this fungal pathogen from different anatomical compartments of sea cucumber (Holothuroidea), including the epidermis, stomach, intestinal tract, and intestinal contents. At present, biological control of sugarcane smut is still in the process of screening and identifying antagonistic bacteria and evaluating their control effects in pot experiments, and it has not yet been applied in the field. Therefore, further research on the biological control of sugarcane smut is necessary.

In this study, we collected rhizosphere soil samples from smut-resistant sugarcane cultivars (‘CP89-2143’ and ‘Yin 8’) at the Sugarcane Breeding Base of South China Agricultural University (SCAU). Bacterial isolates were screened for their ability to effectively inhibit the sexual mating of *S*. *scitamineum* and identified them using 16S rRNA-based taxonomic sequencing. Selected strains underwent comprehensive evaluation through pot experiments, field inoculation trials, and natural field infection tests to determine their disease suppression efficacy and impacts on sugarcane physiological traits, yield, and quality. It addresses the limitation of previous studies in the same field, which focused only on screening biocontrol strains with little attention to field efficacy, thereby providing effective strains and technical support for the practical application of biocontrol against sugarcane smut in production settings.

## 2. Materials and Methods

### 2.1. Experimental Materials

The rhizosphere soil samples used for bacterial isolation were collected from sugarcane cultivars (CP89-2143 and Yin 8) at the Sugarcane Breeding Base of SCAU. Wild-type *S. scitamineum* strains of both MAT-1 and MAT-2 mating types were previously isolated, characterized, and maintained by our laboratory (SCAU Sugarcane Research Group). Teliospores of *S. scitamineum* were obtained from field-infected stalks of susceptible cultivars ‘ROC22’ and ‘LC05-136’ at the Sugarcane Breeding Base of SCAU. WGA-AF488 and propidium iodide staining solutions were purchased from Guangzhou Zhaotian Biotechnology Co., Ltd., Guangzhou, China. For pot experiments and field inoculation trials, smut-susceptible sugarcane cultivars (ROC22 and LC05-136) were used as test materials. Field evaluations under natural infection conditions were conducted using smut-susceptible sugarcane cultivars (‘GT42’ and ‘KY1’). The culture medium was prepared as described by Li [21].

### 2.2. Isolation, Screening, and Identification of Antagonistic Bacteria Against S. scitamineum

#### 2.2.1. Isolation of Antagonistic Bacteria Against *S. scitamineum*

Rhizosphere soil samples were collected from CP89-2143 and Yin 8 at the Sugarcane Breeding Base of SCAU using the standardized five-point sampling method. At each sampling point, soil from 0–10 cm depth was carefully collected from sugarcane roots. After gently shaking off loosely adhered soil, the tightly root-adhering rhizosphere soil (typically 1–2 mm from root surfaces) was collected using sterile brushes. To prepare soil suspensions, 5 g of rhizosphere soil was mixed with 45 mL of sterile water in a conical flask and shaken at 280 rpm for 2 min at 28 °C. Subsequent treatments were performed according to the method described by Chen et al. [22].

#### 2.2.2. Screening of Antagonistic Bacteria Against *S. scitamineum*

On solid YePS medium, 1 μL spots of wild-type *S. Scitamineum* haploid strains of opposite mating types (MAT-1 and MAT-2) were inoculated at 1 cm intervals on both left and right sides. On the left side, the inoculation sites were surrounded by 10 μL of the rhizobacterial suspension (OD_600_ = 1.0) isolated from sugarcane. On the right side, the fungal inoculation sites received no bacterial treatment and served as controls. For each rhizobacterial isolate, three replicate plates were prepared. All plates were sealed and incubated at 28 °C for 2–3 days. The plates were observed for the growth of white mycelia of *S. scitamineum* on both the left and right sides to determine whether the tested bacterium had an antagonistic effect against *S. scitamineum*. In the wild-type control, white fluffy mycelia grew normally. If the *S. scitamineum* colonies surrounded by the tested bacterium failed to produce white fluffy mycelia, or produced significantly weakened white fluffy mycelia, the tested bacterium was identified as an antagonistic bacterium against *S. scitamineum*.

#### 2.2.3. Identification of Antagonistic Bacteria Against *S. scitamineum*

Genomic DNA of antagonistic bacteria was extracted using the cetyltrimethylammonium bromide (CTAB) method following the protocol established by Murray and Thompson [23]. Polymerase chain reaction (PCR) amplification was performed using the primers and cycling conditions detailed in Table S1. The PCR products were subsequently sequenced by Sangon Biotech (Shanghai) Co., Ltd., China (Guangzhou Branch), Guangzhou, China. Sequence analysis of the antagonistic bacteria was conducted based on sequencing data, including BLAST (version 2.17.0) comparisons against the NCBI database and multiple sequence alignment using ClustalW. Strain 2143-2 consisted of 15 sequences and 1570 aligned positions, strain 2143-4 consisted of 14 sequences and 1549 aligned positions, strain 2143-6 consisted of 8 sequences and 1558 aligned positions, strain Y8-2 consisted of 15 sequences and 1568 aligned positions, and strain Y8-3 consisted of 20 sequences and 1549 aligned positions, and was used for phylogenetic tree construction employing the neighbor-joining method in MEGA X (version 10.1.8). The 16S rRNA gene sequences obtained in this study have been deposited in the GenBank database under accession numbers PZ498160–PZ498164.

### 2.3. Antimicrobial Spectrum Test

To determine the antimicrobial spectrum of the biocontrol bacteria, inhibition assays were conducted against the following major sugarcane fungal pathogens preserved in our laboratory: *Nigrospora sphaerica* (sugarcane leaf spot), *Colletotrichum falcatum* (sugarcane red rot), *Bipolaris setariae* (sugarcane brown stripe), *Curvularia ischaemi* (sugarcane ring spot), and *Fusarium verticillioides* (sugarcane pokkah boeng). Biocontrol bacteria were activated and cultured to an OD_600_ of 0.8 and maintained at room temperature for subsequent use. Fungal pathogens were pre-cultured at 28 °C for 7 days, with *B*. *setariae* and *C*. *ischaemi* on solid OA medium and *N*. *sphaerica*, *C*. *falcatum*, and *F*. *verticillioides* were grown on solid PDA medium. Using a sterile punch, fungal agar plugs (5 mm in diameter) were taken from the colony margin and used to inoculate the center of the corresponding culture medium (Petri dish diameter: 90 mm). Simultaneously, sterile filter paper discs (5 mm in diameter) was placed symmetrically at 30 mm intervals on both sides of the fungal plug. Then, 10 μL of the biocontrol bacterial suspension was applied to the filter paper discs, and the control group received 10 μL of liquid LB medium. Each treatment was repeated three times, and the plates were incubated at 28 °C. When the control group colonies fully covered the Petri plate, the inhibition zones in the treatment groups were observed and recorded. The inhibition width and colony diameters were measured, and the mycelial growth inhibition rate was calculated. The inhibition width was the distance between the edge of the antagonistic bacterial colony and the edge of the pathogenic fungal colony. The colony diameter was measured along the axis connecting the center points of the filter paper disc and the fungal plug. The inhibition rate was calculated using the following formula: Inhibition rate (%) = [(Control colony diameter − Treated colony diameter)/(Control colony diameter − Fungal plug diameter)] × 100%.

### 2.4. Visualization of S. scitamineum Hyphal Invasion in Sugarcane Buds

The experiment was conducted using two smut-susceptible sugarcane cultivars, ROC22 and LC05-136, as test materials. The following seven treatments were established: CK1 (sugarcane buds soaked in sterile water), CK2 (sugarcane buds soaked in *S*. *scitamineum* teliospores), T1 (CK2 + strain 2143-2), T2 (CK2 + strain 2143-4), T3 (CK2 + strain 2143-6), T4 (CK2 + strain Y8-2), and T5 (CK2 + strain Y8-3). For each treatment, 20 healthy, fresh sugarcane buds were selected per cultivar. CK1 sugarcane buds were soaked in sterile water for 30 min, and those in CK2 were soaked in a *S*. *scitamineum* teliospore suspension (5 × 10^6^ spores/mL) for 30 min. For treatments T1, T2, T3, T4, and T5, the sugarcane buds were first immersed in a bacterial suspension (OD_600_ = 0.8) of the antagonistic strains for 6 h and then soaked in a *S*. *scitamineum* teliospore suspension (5 × 10^6^ spores/mL) for an additional 30 min. The samples (sugarcane buds) were cultured in an incubator with light at 28 °C and collected at 2, 4, 6, 9, and 12 days post treatment for analysis. The collected specimens were co-stained with WGA-AF488 and propidium iodide following the manufacturer’s protocol. The hyphal growth of *S*. *scitamineum* teliospores in the sugarcane buds under different treatments was observed and photographed using an inverted fluorescence microscope (Leica DMi8, (Leica DMi8, Leica Microsystems GmbH, Wetzlar, Germany)).

### 2.5. Biocontrol Efficacy Evaluation of Antagonistic Bacteria Against Sugarcane Smut in Pot Experiments

The experimental treatments followed the same design as described in Section 2.4. Antagonistic bacterial strains were first activated by culturing on solid LB medium for 24–48 h. Single colonies were used to inoculate liquid LB medium and incubated in a shaker at 28 °C with agitation at 200 rpm until reaching an OD_600_ of 0.8. Healthy and plump single-bud cuttings of sugarcane cultivars ROC22 and LC05-136 were first soaked in the antagonistic bacterial suspension for 6 h. After removal, the cuttings were shade-dried at an ambient temperature. Subsequently, they were immersed in a *S*. *scitamineum* teliospore suspension (5 × 10^6^ spores/mL) for 30 min. Following treatment, the cuttings were incubated at 28 °C for 24 h to promote bud sprouting. The prepared cuttings were then planted in pots, with five sugarcane buds per pot and three pots per replicate. Each treatment consisted of three replicates. The experiment was conducted in the greenhouse at the Sugarcane Breeding Base of SCAU, and planting was initiated on 6 March 2023. Standard sugarcane growth management practices were followed throughout the cultivation period.

### 2.6. Field Evaluation of the Biocontrol Efficacy Against Sugarcane Smut

To confirm the results of the pot-based biocontrol experiments, field inoculation trials were conducted using the most effective antagonistic strains: T2 (2143-4, *Bacillus subtilis*), T3 (2143-6, *Burkholderia diffusa*), and T4 (Y8-2, *Pseudomonas reinekei*). The test cultivars and the strain activation and inoculation procedures followed the same protocols described in Section 2.5. The experiment was established on 25 March 2024, at the Sugarcane Breeding Base of SCAU. A randomized complete block design was adopted, with experimental plots having a 1 m row length, 1 m row spacing, and 3 rows per plot. Each row was planted with 10 single-bud cuttings, resulting in 30 cuttings per plot. There were three replicates per treatment. Fertilization consisted of 600 kg/ha of 15-15-15 compound fertilizer (produced by Guangzhou Ladomei Co., Ltd., Guangzhou, China). To avoid potential effects on the smut fungus and biocontrol agents, 3% phoxim granules were not applied for insect control, and plastic mulch was not used. All other field management practices followed local sugarcane production standards. The disease incidence and control efficacy of sugarcane smut were determined according to the methodology described in Section 2.8.1.

### 2.7. Field Evaluation of Biocontrol Efficacy Under Natural Infection Conditions

Natural infection trials were conducted during the initial outbreak period of sugarcane smut in April 2025 to evaluate the field control efficacy of the selected biocontrol strains. The experiments were carried out at three major sugarcane-producing farms belonging to the Zhanjiang Agricultural Reclamation Bureau of Guangdong Province in China’s primary sugarcane cultivation region: Torch Farm (20°46′8.515″ N, 109°56′39.990″ E), Golden Star Farm (20.69° N, 110.03° E), and Huguang Farm (ranging from 21°2′30″ N to 21°10′8″ N and 110°12′37″ E to 110°20′44″ E). A comparative field trial was conducted by dividing the naturally infected experimental fields planted with susceptible sugarcane cultivars into two groups, control and treatment, each covering an area of 300 m^2^. The procedures for strain activation and preparation of bacterial suspensions followed the same protocols described in Section 2.5. The biocontrol strains were uniformly sprayed onto sugarcane leaves at an application rate of 54 L per treatment group, and an equivalent volume of sterile water was applied to the control group. Two applications were performed on 9 April and 27 April 2025. The disease incidence was assessed on 28 May 2025, in 3 representative rows (20 m row length per replicate) selected from both treatment and control groups to determine the sugarcane smut incidence. Control efficacy was calculated according to the methodology detailed in Section 2.8.1.

### 2.8. Assessment of Sugarcane Smut Incidence and Physiological Parameters

#### 2.8.1. Sugarcane Smut Incidence and Latent Period

The latent period of sugarcane smut was defined as the number of days from inoculation to the initial emergence of smut whips. Following disease onset, the smut incidence was monitored weekly for 6 months. To prevent teliospore spread, all plants with smut were bagged immediately after investigation. The number of diseased plants was recorded in each survey, and the cumulative data were used to calculate both the disease incidence and control efficacy. The disease incidence was calculated using the following formula: Disease incidence (%) = (Number of infected plants/Total number of plants) × 100%. The control efficacy was determined as follows: Control efficacy (%) = [(Incidence of plants inoculated only with *S*. *scitamineum* teliospores − Treatment incidence)/Incidence of plants inoculated only with *S*. *scitamineum* teliospores] × 100% [24].

#### 2.8.2. Determination of Physiological Parameters

In the pot experiment, fresh +1 leaves were collected at three critical growth stages (seedling, tillering, and jointing stages) for comprehensive antioxidant profiling. The collected leaf samples were immediately flash-frozen in liquid nitrogen and stored at −80 °C for subsequent quantification of key antioxidant system components: superoxide dismutase (SOD) activity, catalase (CAT) activity, peroxidase (POD) activity, the rate of superoxide anion (O_2_^−^) production, hydrogen peroxide (H_2_O_2_) content, and malondialdehyde (MDA) content.

### 2.9. Sugarcane Brix and Cane Yield in the Pot Experiment

At maturity (10 November 2023), the sugar Brix was determined in the middle of the sugarcane stem in the pot experiment using a hand-held field sugar Brix meter (ATAGO N1, ATAGO Co., Ltd., Tokyo, Japan). Five representative stalks per treatment were randomly selected for Brix measurement, followed by harvesting to determine the stalk yield per pot.

### 2.10. Data Processing and Analysis

Raw data were processed using Microsoft Excel 2010, and graphs were generated using GraphPad Prism 8.3. Statistical analyses were performed using IBM SPSS Statistics 27. For all data except those presented in Table 4, one-way analysis of variance (ANOVA) was first conducted on samples from different groups. If significant differences were detected (*p* < 0.05), the LSD-*t* test was further used for multiple comparisons among sample means. For the data presented in Table 4, the independent samples *t*-test (Student’s *t*-test) was used to determine whether significant differences existed between the means of the two groups.

## 3. Results

### 3.1. Screening and Identification of Antagonistic Bacteria Against S. scitamineum

#### 3.1.1. Screening of Antagonistic Bacteria Against *S. scitamineum*

**Figure 1:**
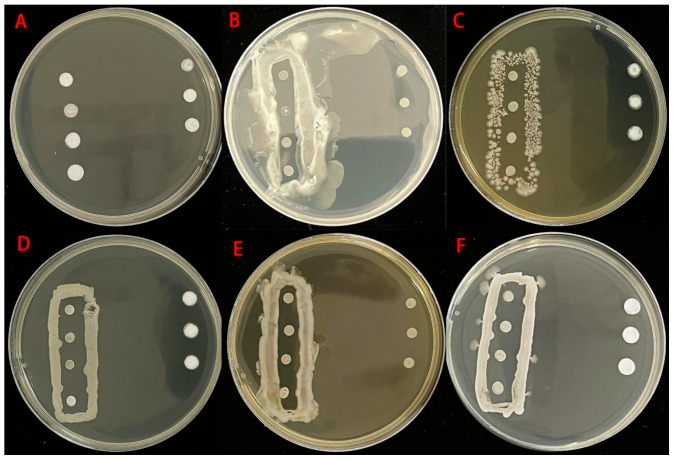
Screening of antagonistic bacteria against *S*. *scitamineum*. (**A**). *S*. *scitamineum* (CK: control); (**B**). strain 2143-2; (**C**). strain 2143-4; (**D**). strain 2143-6; (**E**). strain Y8-2; (**F**). strain Y8-3.

**Figure 2:**
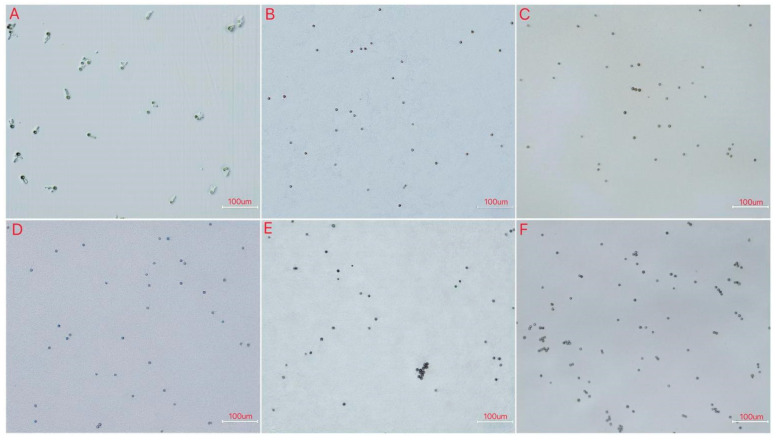
Inhibitory effect of antagonistic bacteria on *S*. *scitamineum* teliospore germination. (**A**). CK; (**B**). Y8-2; (**C**). Y8-3; (**D**). 2143-2; (**E**). 2143-4; (**F**). 2143-6.

A total of 273 bacterial strains were isolated and purified from solid LB medium. Through systematic screening, five strains exhibiting significant antagonistic activity against *S*. *scitamineum* were identified (Figure 1) and designated as 2143-2, 2143-4, 2143-6, Y8-2, and Y8-3. The inhibition rates of these five antagonistic strains on *S*. *scitamineum* teliospores were 89.83, 80.85, 88.46, 94.29, and 81.82%, respectively (Figure 2).

#### 3.1.2. Molecular Identification of Antagonistic Bacteria

**Figure 3:**
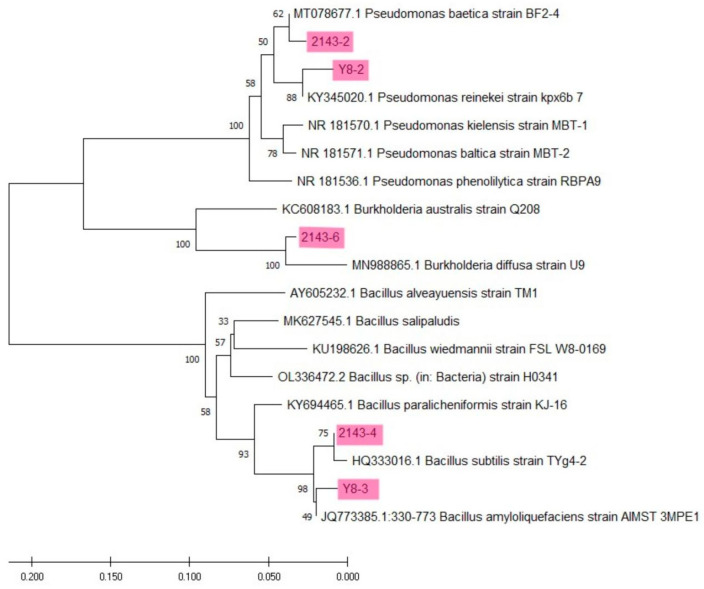
Phylogenetic tree of antagonistic bacterial strains based on 16S rRNA gene sequences using the neighbour-joining method. Strains marked in red represent the bacterial isolates obtained in this study.

To amplify the 16S rRNA sequence, primer pair 27F/1492R was used for strain 2143-2, and primer pair 338F/806R primer was used for the other four strains (2143-4, 2143-6, Y8-2, and Y8-3). The PCR products were detected using electrophoresis to obtain the amplification products of the expected size, and the sequences were sequenced and compared by BLAST using NCBI. The phylogenetic tree was constructed using MEGA-X software (version 10.1.8). Strains 2143-2, 2143-4, 2143-6, Y8-2, and Y8-3 were highly similar to *Pseudomonas baetica*, *B*. *subtilis*, *B*. *diffusa*, *P*. *reinekei* and *Bacillus amyloliquefaciens*, respectively (Figure 3 and Figure S1).

### 3.2. Antimicrobial Spectrum of Biocontrol Strains

**Figure 4:**
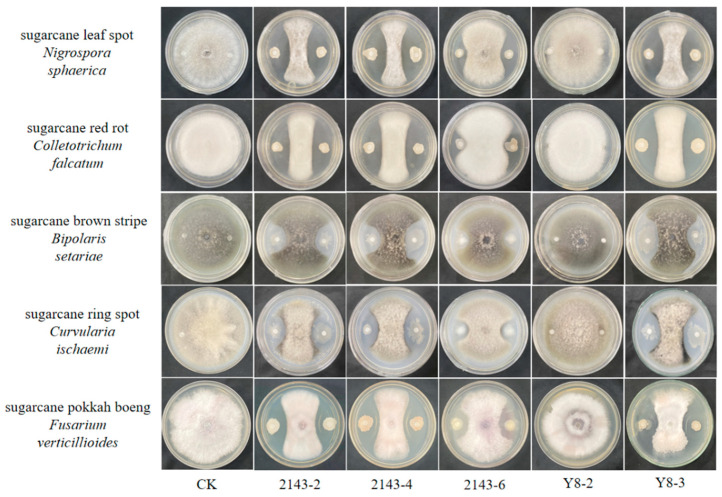
Antagonistic effect of biocontrol bacteria against sugarcane pathogens.

As shown in Figure 4 and Table 1, biocontrol strains 2143-2, 2143-4, and Y8-3 exhibited strong antagonistic activity against all five tested sugarcane fungal pathogens. Strain 2143-2 exhibited inhibition rates of 77.16% against *N*. *sphaerica* (sugarcane leaf spot), 72.55% against *C*. *falcatum* (sugarcane red rot), 74.51% against *B*. *setariae* (sugarcane brown stripe), 70.98% against *C*. *ischaemi* (sugarcane ring spot), and 66.27% against *F*. *verticillioides* (sugarcane pokkah boeng). Strain 2143-4 showed inhibition rates of 76.53, 69.41, 72.16, 70.20, and 70.59%, respectively. Strain Y8-3 demonstrated inhibition rates of 69.78, 68.24, 70.98, 73.14, and 71.37%, respectively. Strain 2143-6 demonstrated limited antifungal activity against *F*. *verticillioides* (sugarcane pokkah boeng) with an inhibition rate of 20.00% but moderately suppressed (50.98–59.76%) the other four tested sugarcane pathogens. In contrast, strain Y8-2 showed minimal inhibitory effects (0–7.45%) across all five pathogens.

### 3.3. Visualization of S. scitamineum Hyphal Invasion in Sugarcane Buds

**Figure 5:**
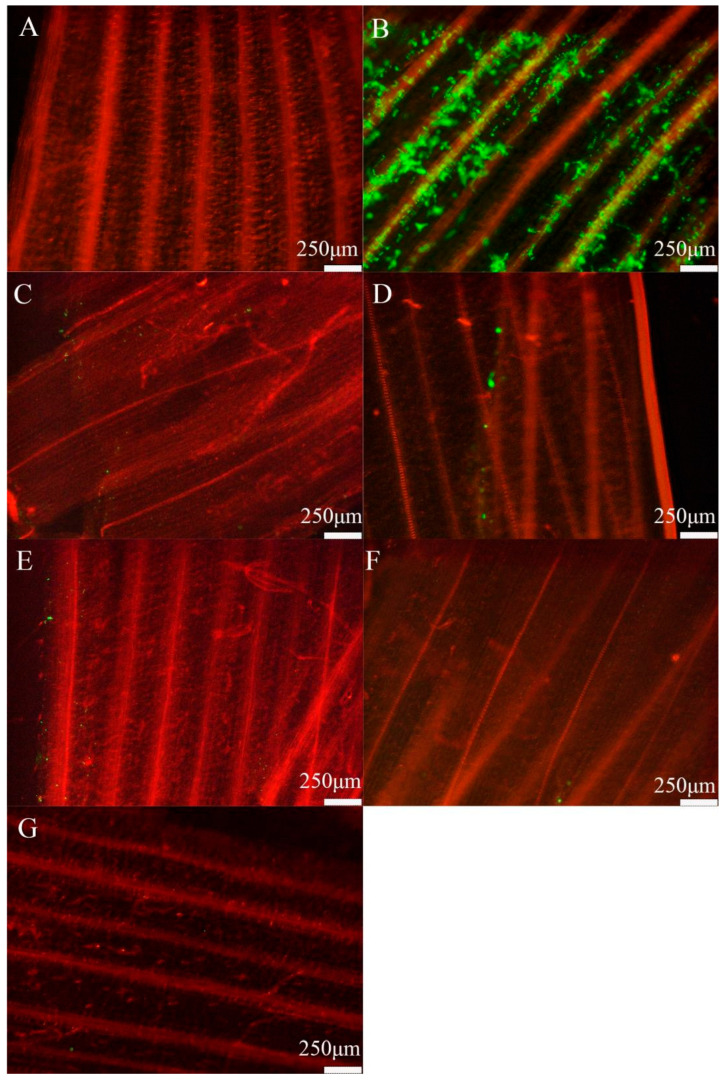
Mycelium produced by the germination of *S*. *scitamineum* teliospores infecting ROC22 sugarcane buds (observation at 100 times magnification at 6 days after infection). (**A**). CK1 (sterile water control); (**B**). CK2 (teliospore suspension only); (**C**). T1 (CK2 + strain 2143-2); (**D**). T2 (CK2 + strain 2143-4); (**E**). T3 (CK2 + strain 2143-6); (**F**). T4 (CK2 + strain Y8-2); (**G**). T5 (CK2 + strain Y8-3).

**Figure 6:**
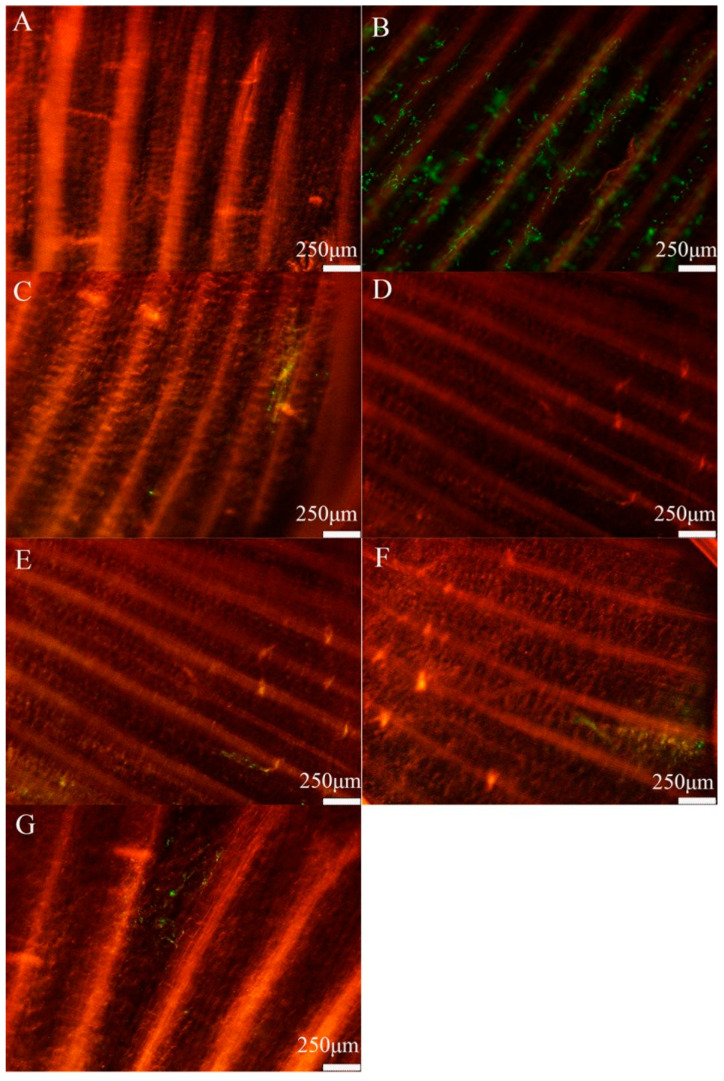
Mycelium produced by the germination of *S*. *scitamineum* teliospores infecting LC05-136 sugarcane buds (observation at 100 times magnification at 9 days after infection). (**A**). CK1 (sterile water control); (**B**). CK2 (teliospore suspension only); (**C**). T1 (CK2 + strain 2143-2); (**D**). T2 (CK2 + strain 2143-4); (**E**). T3 (CK2 + strain 2143-6); (**F**). T4 (CK2 + strain Y8-2); (**G**). T5 (CK2 + strain Y8-3).

For the ROC22 cultivar, no green fluorescent hyphae were detected in CK1 during the 12-day observation period. In contrast, CK2 exhibited initial colonization of fluorescent hyphae at 4 days post inoculation, with progressive increases in both hyphal density and length observed during subsequent sampling time points. Sugarcane buds inoculated with antagonistic bacterial strains 2143-2, 2143-4, and 2143-6 first showed green fluorescent hyphae on day 6 but with a significantly lower hyphal density and shorter hyphal length than CK2 (Figure 5). In contrast, buds treated with antagonistic strains Y8-2 and Y8-3 showed delayed fungal colonization, with green fluorescent hyphae not detected until day 9, and hyphal growth was similarly suppressed. For the LC05-136 cultivar, no green fluorescent hyphae were detected in CK1 during the 12-day observation period. CK2 exhibited initial fungal colonization with fluorescent hyphae observed on day 4. Sugarcane buds treated with antagonistic bacteria 2143-2, 2143-6, Y8-2, and Y8-3 showed delayed hyphal emergence (day 9) (Figure 6), with a significantly reduced hyphal density and shorter hyphal length than CK2. Strain 2143-4 had the strongest suppression effect, with fluorescent hyphae not detected until day 12, showing the largest reductions in both hyphal parameters. These results indicate that all tested bacterial strains differentially inhibited teliospore germination, hyphal formation, and *S*. *scitamineum* development, with strain-specific variation in suppression efficacy.

### 3.4. Biocontrol Efficacy

#### 3.4.1. Biocontrol Efficacy Based on Pot Experiment

**Figure 7:**
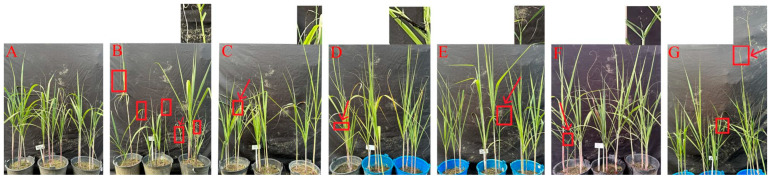
Sugarcane smut symptoms in sugarcane cultivar ROC22. (**A**). CK1 (sterile water control); (**B**). CK2 (teliospore suspension only); (**C**). T1 (CK2 + strain 2143-2); (**D**). T2 (CK2 + strain 2143-4); (**E**). T3 (CK2 + strain 2143-6); (**F**). T4 (CK2 + strain Y8-2); (**G**). T5 (CK2 + strain Y8-3). Red arrows point to black whips of sugarcane smut.

**Figure 8:**
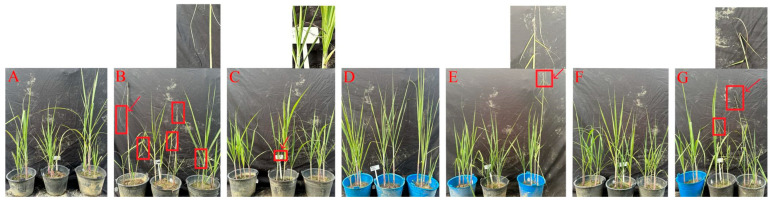
Sugarcane smut symptoms in sugarcane cultivar LC05-136. (**A**). CK1 (sterile water control); (**B**). CK2 (teliospore suspension only); (**C**). T1 (CK2 + strain 2143-2); (**D**). T2 (CK2 + strain 2143-4); (**E**). T3 (CK2 + strain 2143-6); (**F**). T4 (CK2 + strain Y8-2); (**G**). T5 (CK2 + strain Y8-3). Red arrows point to black whips of sugarcane smut.

No smut symptoms were observed in either sugarcane cultivar under CK1 (water control) treatment. CK2 (teliospore-soaking) resulted in the highest disease incidence (Table 2; Figure 7 and Figure 8) at 66.67%, with the shortest latent period (106 days) and no control efficacy. All biocontrol bacterial treatments significantly reduced smut incidence compared to CK2, prolonged the latent period beyond 106 days, and achieved statistically significant control efficacy. For sugarcane cultivar ROC22, application of biocontrol bacterial treatments resulted in a 38.89–58.34% reduction in smut disease incidence and a 58.37–87.51% improvement in control efficacy (Table 2). No statistically significant differences were observed among the biocontrol treatments in terms of either disease incidence or control efficacy. For sugarcane cultivar LC05-136, the application of biocontrol bacterial treatments resulted in a 38.89–66.67% reduction in smut disease incidence and a 58.37–100% improvement in control efficacy. Among all treatments, inoculation with strains 2143-4 (T2 treatment) and Y8-2 (T4 treatment) resulted in the lowest disease incidence in both sugarcane cultivars. These two strains demonstrated a control efficacy of 87.51% in ROC22 and 100% in LC05-136. Furthermore, in ROC22, these treatments exhibited the longest disease latent period, showing a 35-day delay compared to CK2 (Table 2).

#### 3.4.2. Biocontrol Efficacy Based on the Field Inoculation Experiment

No smut symptoms were observed in either sugarcane cultivar under CK1. Although varying degrees of disease incidence occurred under both CK2 and the biocontrol bacterial treatments, all biocontrol bacterium applications demonstrated statistically significant reductions in disease incidence compared to CK2. T4 demonstrated the lowest smut incidence, showing significant reductions compared to T2 and T3. T2 exhibited lower disease incidence than T3, but the difference was not statistically significant. Similar trends were observed for disease control efficacy. T4 exhibited optimal biocontrol performance, achieving 68.40 and 73.99% control efficacy in ROC22 and LC05-136, respectively, with statistically superior outcomes compared to T2 and T3. Although T2 showed a marginally higher efficacy than T3, the difference was not statistically significant (Table 3).

#### 3.4.3. Biocontrol Efficacy Under Natural Field Infection Conditions

The incidence rates of sugarcane smut in the treatment groups of GT42 at Torch Farm and KY1 at Huguang Farm (sprayed with biocontrol strain 2143-4 or Y8-2) were 7.69 and 5.75%, respectively, which were significantly lower than those in the control group (sprayed with water), and the control effects reached 59.01 and 65.73%, respectively, reaching significant levels. At Golden Star Farm, GT42 plots treated with strain 2143-6 and the water control exhibited low disease incidence (2.00% vs. 2.67%). Although the treatment group had a reduced incidence, the difference was not statistically significant, achieving 25.09% control efficacy (Table 4).

**Table 4:** Disease incidence and control efficacy of sugarcane smut under natural field infection conditions.

Farm	Cultivar	Plant Type	Treatment	Disease Incidence (%)				Control Efficacy (%)
				I	II	III	Average	
Torch Farm	GT42	Second-year ratoon	Water	10.83	25.00	20.45	18.76	0.00 ± 0.00
			2143-4	5.37	8.62	9.09	7.69 *	59.00 ± 10.81 *
Golden Star Farm	GT42	First-year ratoon	Water	2.00	3.00	3.00	2.67	0.00 ± 0.00
			2143-6	0.00	3.00	3.00	2.00	25.09 ± 64.95
Huguang Farm	KY1	First-year ratoon	Water	12.82	16.04	21.48	16.78	0.00 ± 0.00
			Y8-2	6.96	6.14	4.17	5.75 *	65.73 ± 8.55 *

Note: (*) indicates that the difference between the treatment group and the control group for the same variety was statistically significant (*p* < 0.05).

### 3.5. Antioxidant Enzyme Activities and Oxidative Product Contents in Sugarcane Leaves

**Figure 9:**
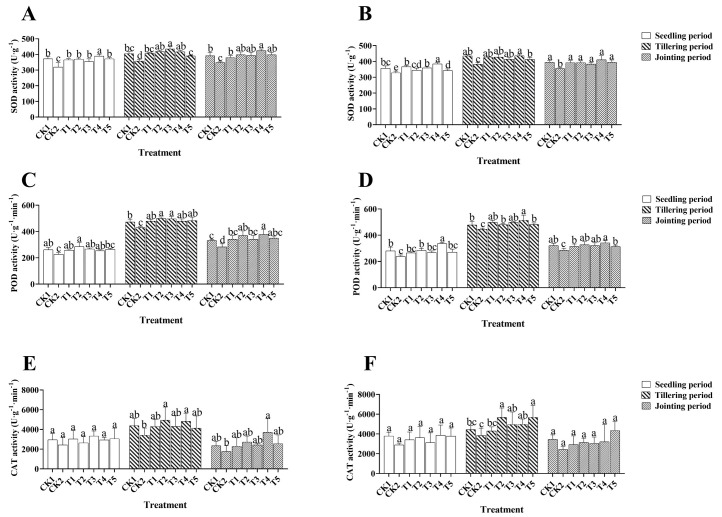
Peroxidase (POD), superoxide dismutase (SOD), and catalase (CAT) activities in sugarcane leaves. (**A**). SOD activity in cultivar ROC22; (**B**). SOD activity in cultivar LC05-136; (**C**). POD activity in cultivar ROC22; (**D**). POD activity in cultivar LC05-136; (**E**). CAT activity in cultivar ROC22; (**F**). CAT activity in cultivar LC05-136. Bars represent the standard error of three independent replicates in each group, and different lowercase letters represent a significant difference at the 0.05 level.

At the three sugarcane growth stages, the SOD activity was significantly lower in the ROC22 cultivar under CK2 than under the other treatments. At the seedling stage, T1, T2, T3, and T5 showed no significant differences in SOD activity compared to the non-inoculated control (CK1); however, it was significantly higher under T4 than under CK1. At the tillering stage, only T3 demonstrated a significantly higher SOD activity than CK1, whereas the other four antagonistic bacterial treatments (T1, T2, T4, and T5) showed no significant differences from CK1. At the jointing stage, the SOD activity was again significantly higher under T4 than under CK1, while that of the remaining antagonistic bacterial treatments (T1, T2, T3, and T5) did not differ significantly from the control. CK2 consistently exhibited the lowest POD activity across all three growth stages. At the seedling stage, none of the antagonistic bacterial treatments (T1–T5) showed significant differences in POD activity compared to CK1. Although T5 did not differ significantly from CK2, all other bacterial treatments (T1–T4) had a significantly higher POD activity than CK2. At the tillering stage, treatments T2 and T3 had a significantly elevated POD activity compared to CK1, whereas the remaining bacterial treatments (T1, T4, and T5) showed no significant differences from CK1. Notably, all antagonistic bacterial treatments exhibited a significantly higher POD activity than CK2. At the jointing stage, treatments T1, T3, and T5 did not differ significantly from CK1, whereas T2 and T4 had a significantly higher POD activity than CK1. As observed in previous stages, all bacterial treatments maintained a significantly higher POD activity than CK2. CK2 consistently had the lowest CAT activity among all treatments across the three growth stages. At the seedling stage, no significant differences in CAT activity were observed among the treatments. At the tillering stage, none of the antagonistic bacterial treatments (T1–T5) exhibited significant differences from CK1. However, treatments T2 and T4 had a significantly higher CAT activity than CK2, while the remaining treatments showed no significant differences from CK2. At the jointing stage, all antagonistic bacterial treatments maintained CAT activity levels comparable to CK1. Only T4 showed a significantly elevated activity compared to CK2, with other treatments displaying no statistical difference from CK2. At the seedling stage, all antagonistic bacterial treatments (T1–T5) exhibited significantly elevated O_2_^−^ production compared to CK2. Although T1 showed no significant difference from CK1, the remaining treatments (T2–T5) demonstrated significantly higher production rates than CK1. At the tillering stage, T4 displayed significantly increased O_2_^−^ production compared to both CK1 and CK2. No other antagonistic bacterial treatments (T1–T3 and T5) showed statistically significant differences from either control group. At the jointing stage, T3 and T5 maintained significantly higher production rates compared to both CK1 and CK2. The other bacterial treatments (T1, T2, and T4) did not differ significantly from either control. At the seedling and tillering stages, CK2 showed significantly higher H_2_O_2_ levels than all other treatments. At the jointing stage, the H_2_O_2_ content of CK2 was also significantly higher than that of CK1, T1, T2, and T4, and there were no significant differences among CK2, T3, and T5. There were no significant differences between T3 and CK1 at the seedling stage, and other treatments inoculated with antagonistic bacteria showed significantly higher values than CK1. T2, T3, and T4 were not significantly different from CK1 at the tillering stage, but T1 and T5 had significantly higher values than CK1. T1, T2, and T4 showed no significant difference from CK1 at the jointing stage, but T3 and T5 had significantly higher values than CK1 (Figure 9).

**Figure 10:**
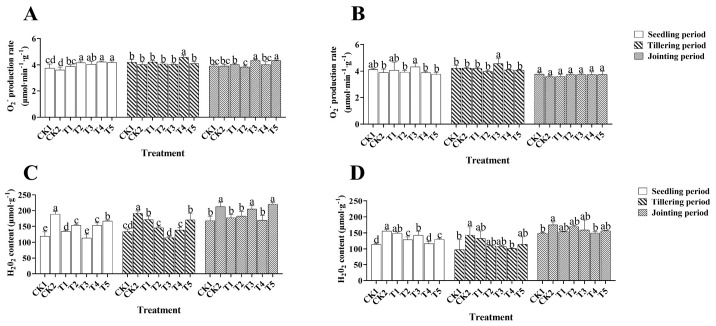
Superoxide anion (O_2_^−^) production and hydrogen peroxide (H_2_O_2_) content in sugarcane leaves. (**A**). O_2_^−^ production in cultivar ROC22; (**B**). O_2_^−^ production in cultivar LC05-136; (**C**). H_2_O_2_ content in cultivar ROC22; (**D**). H_2_O_2_ content in cultivar LC05-136. Bars represent the standard error of three independent replicates in each group, and different lowercase letters represent a significant difference at the 0.05 level.

At the three sugarcane growth stages, LC 05-136 under CK2 showed the weakest SOD activity, which was significantly lower than that of the other treatments. At the seedling stage, T1, T2, and T3 showed no significant differences in SOD activity compared to CK1. T4 demonstrated a significantly higher SOD activity than CK1, whereas that of T5 was significantly lower than that of CK1. At the tillering and jointing stages, none of the antagonistic bacterial treatments (T1–T5) exhibited statistically significant differences in SOD activity compared to CK1. CK2 consistently demonstrated the lowest POD activity among all treatments during the three examined growth stages. At the seedling stage, T1, T2, T3, and T5 showed no significant differences in POD activity compared to CK1. T4 exhibited a significantly higher POD activity than CK1. Although T1, T3, and T5 did not differ significantly from CK2, both T2 and T4 displayed a significantly elevated activity compared to CK2. At the tillering stage, only T4 maintained a significantly higher POD activity than CK1. The remaining bacterial treatments (T1–T3 and T5) showed comparable activity to CK1. All antagonistic bacterial treatments had a significantly greater POD activity than CK2. The results at the jointing stage were similar to those at the tillering stage. CK2 consistently exhibited the lowest CAT activity among all treatments at the three growth stages examined. At the seedling and jointing stages, no statistically significant differences in CAT activity were observed among treatments. At the tillering stage, T1, T3, and T4 maintained CAT activity levels comparable to CK1. Both T2 and T5 demonstrated significantly enhanced CAT activity relative to CK1. Except for T1, which showed no difference from CK2, all antagonistic bacterial treatments (T2–T5) exhibited a significantly higher CAT activity than CK2. At the seedling stage, no significant differences in O_2_^−^ production were observed between any of the antagonistic bacterial treatments (T1–T5) and CK1. T3 had a significantly elevated O_2_^−^ production compared to CK2, while other treatments maintained levels similar to CK2. At the tillering stage, T3 had a significantly higher O_2_^−^ production than both CK1 and CK2. The remaining antagonistic bacterial treatments (T1, T2, T4, and T5) exhibited no statistically significant differences from either control group. At the jointing stage, all bacterial treatments showed O_2_^−^ production rates comparable to CK1. T4 and T5 displayed significantly increased generation compared to CK2. Other treatments (T1–T3) did not differ significantly from CK2. CK1 consistently showed the lowest H_2_O_2_ content across all three growth stages. CK2 maintained the highest H_2_O_2_ content throughout the experimental period. At the seedling stage, all antagonistic bacterial treatments except T4 exhibited a significantly higher H_2_O_2_ content than CK1. Only T1 showed no significant difference from CK2, with other treatments (T2–T5) having significantly lower accumulation than CK2. At the tillering and jointing stages, no significant differences were observed between any antagonistic bacterial treatments and CK1. T4 had a significantly reduced H_2_O_2_ content compared to CK2. The remaining treatments (T1–T3 and T5) maintained an H_2_O_2_ content statistically comparable to CK2 (Figure 10).

### 3.6. Malondialdehyde (MDA) Content in Sugarcane Leaves

**Figure 11:**
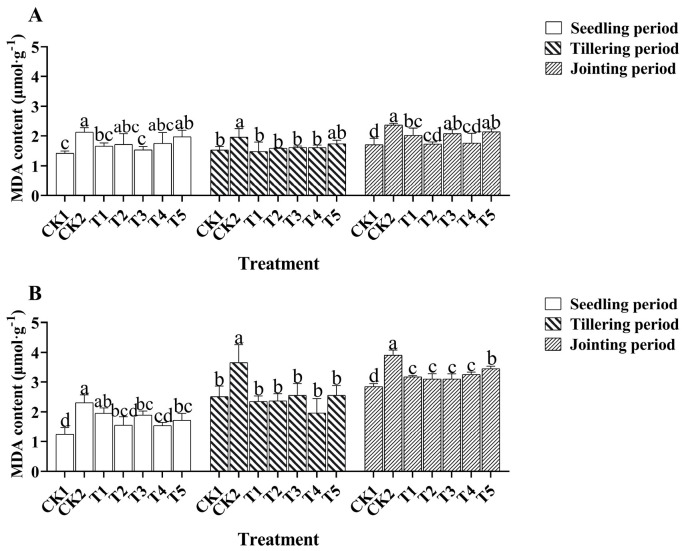
Malondialdehyde (MDA) content in sugarcane leaves. (**A**). MDA content in cultivar ROC22; (**B**). MDA content in cultivar LC05-136. Bars represent the standard error of three independent replicates in each group, and different lowercase letters represent a significant difference at the 0.05 level.

For the ROC22 cultivar, there were no significant differences in MDA content between treatments with antagonistic bacteria and CK1 at the seedling and tillering stages (except under T5 at the seedling stage, which had a content significantly higher than that under CK1). At the jointing stage, the MDA content was significantly higher under T1, T3, and T5 than under CK1, but there was no significant differences among T2, T4, and CK1. The MDA content was highest under CK2 at all three stages, showing values significantly higher than those of T1 and T3 at the seedling stage, T1 at the tillering stage, and T1, T2, and T4 at the jointing stage. There were no significant differences between CK2 and other treatments inoculated with antagonistic bacteria at any of the stages (Figure 11A).

For the LC05-136 cultivar, the MDA content of CK1 was low at all three stages, while that of CK2 was highest. T1, T3, and T5 showed significantly higher values than CK1 at the seedling stage. There were no significant differences among T2, T4, and CK1; however, CK2 had a significantly higher value than the treatments with antagonistic bacteria, except T1, which showed no significant difference from CK2. At the tillering and jointing stages, the MDA content was significantly higher under CK2 than under all treatments inoculated with antagonistic bacteria, and there were no significant differences between treatments inoculated with antagonistic bacteria and CK1 at the tillering stage. All treatments inoculated with antagonistic bacteria showed significantly higher values than CK1 at the jointing stage (Figure 11B).

### 3.7. Sugarcane Brix and Cane Yield in the Pot Experiment

The sugar Brix was significantly reduced in the two tested sugarcane cultivars by inoculation with *S*. *scitamineum* teliospores (CK2) and improved to some extent by inoculation with antagonistic bacteria compared to CK2, but it was still lower than that under CK1. Only LC05-136 under T2 was slightly higher than that under CK1, and the difference was not significant. In addition, for ROC22, the sugar Brix under T1, T3, and T5 was significantly lower than that under CK1, but there were no significant differences among T2, T4, and CK1. For LC 05-136, there were no significant differences between the treatments inoculated with antagonistic bacteria and CK1 (Table 5).

Inoculation with *S*. *scitamineum* teliospores significantly reduced the cane yield of the two sugarcane varieties. Inoculation with antagonistic bacteria improved the cane yield to some extent compared to CK2; however, it was still lower than that of CK1. Only LC 05-136 under T2 had a slightly higher yield than CK1, and the difference was not significant. For ROC22, treatments inoculated with antagonistic bacteria (T1, T2, T3, T4, and T5) showed a significant increase in cane yield by 43.03, 51.19, 49.24, 52.04, and 40.60%, respectively, compared to CK2, but all of them reduced the cane yield compared to CK1. The cane yield of T1 and T5 reached a significant level. For LC 05-136, the treatments inoculated with antagonistic bacteria (T1, T2, T3, T4, and T5) significantly increased the cane yield by 44.63, 78.56, 41.91, 63.40, and 44.58%, respectively, compared to CK2. The cane yield of all treatments except T2 was not significantly higher than that of CK1, and the cane yield of other treatments was lower than that of CK1, with T1, T3, and T5 reaching significant levels (Table 5).

## 4. Discussion

Biological control of plant diseases primarily relies on rhizosphere microorganisms producing compounds that inhibit pathogen growth, thereby preventing or hindering their development. Utilizing rhizosphere microbes for plant disease control avoids significant environmental issues, improves soil properties, enhances fertility, and offers advantages, such as high efficiency [25]. Due to the growing emphasis on green agriculture, biofertilizers have gained increasing attention in recent years. Consequently, biological control of plant diseases has become a prominent research focus, and this trend holds equally true for sugarcane production. Ta [26] isolated 68 bacterial strains from sugarcane rhizosphere soil and found that all strains exhibited sugarcane growth-promoting effects. Seven of these strains showed significant antagonistic activity against sugarcane smut. In this study, we collected sugarcane rhizosphere soil and identified five bacterial strains with significant antagonistic activity against *S*. *scitamineum*: 2143-2 (*P*. *baetica*), 2143-4 (*B*. *subtilis*), 2143-6 (*B*. *diffusa*), Y8-2 (*P*. *reinekei*), and Y8-3 (*B*. *amyloliquefaciens*). Following the methodology described by Redkar et al. [27], who used WGA-AF488 and propidium iodide to visualize the fungal hyphae of *Ustilago maydis* and *U*. *hordei* in maize and barley leaves, respectively, this study employed green fluorescent visualization to observe *S*. *scitamineum* teliospore germination and hyphal development in sugarcane buds. Using pot experiments, field inoculation trials, and natural infection assessments, we confirmed the biocontrol efficacy of antagonistic bacteria against sugarcane smut. Strains 2143-4 (*B*. *subtilis*) and Y8-2 (*P*. *reinekei*) demonstrated superior control effects, emerging as optimal candidates for *S*. *scitamineum* management.

In the study of antagonistic bacteria against sugarcane smut, Ta [26] found that isolated bacterial strains G1 (*Burkholderia cepacia*), G10 (*Burkholderia contaminants*), G12 (*Serratia rubidaea*), and G23 (*Burkholderia gladioli*) exhibited varying inhibitory effects on *S*. *scitamineum*. These strains significantly prolonged the latent period. Strain G23 completely prevented smut occurrence for 220 days post inoculation and promoted sugarcane growth. In our pot experiments, strain 2143-6 (*B*. *diffusa*), which belongs to the same genus as the aforementioned G23 (*B*. *gladioli*), demonstrated significant biocontrol efficacy against sugarcane smut. When applied to cultivar ROC22, it showed a disease incidence of 11.11% and control efficacy of 83.36%. For cultivar LC05-136, the incidence was 16.67% with 75.01% control efficacy, both representing statistically significant reductions in smut occurrence. Jayakumar et al. [28] isolated strain ESR3 (*B*. *amyloliquefaciens*) from sugarcane roots, which showed strong in vitro antagonistic activity against *S*. *scitamineum*, consistent with our findings for strain Y8-3 (*B*. *amyloliquefaciens*) obtained from sugarcane rhizosphere soil. Although Jayakumar et al. [28]’s greenhouse pot experiments demonstrated complete disease prevention (0% incidence over 10 months) using ESR3, our similar experiments showed 27.3% disease incidence with Y8-3. This discrepancy may be attributed to differences in bacterial application protocols, particularly the longer immersion time (12 h vs. our 6 h), which may have enhanced the microbial colonization observed by Jayakumar et al. Additionally, the *Bacillus* spp. strains isolated in this study—2143-4 (*B*. *subtilis*) and Y8-3 (*B*. *amyloliquefaciens*)—represent well-documented biocontrol agents that have broad-spectrum efficacy against plant pathogens beyond sugarcane smut. He et al. [29] demonstrated that *B*. *subtilis* and *B*. *amyloliquefaciens* effectively controlled ginseng rust rot (*Cylindrocarpon destructans*). Inoculation of ginseng rhizomes with these antagonistic bacteria significantly reduced both the disease index and disease incidence. Similarly, Medeiros et al. [30] found that the application of *Bacillus* sp. RAB9 to melon seeds infected with *Acidovorax citrulli* reduced the lesion area by 47% and extended the latent period by 35%. In this study, strains 2143-4 (*B*. *subtilis*) and Y8-3 (*B*. *amyloliquefaciens*) demonstrated significant control efficacy against sugarcane smut. Specifically, 2143-4 (*B*. *subtilis*) showed 87.51 and 100% control efficacy in pot experiments and 62.48 and 69.86% control efficacy in field inoculation trials with cultivars ROC22 and LC05-136, respectively, and 59.01% control efficacy for susceptible cultivar GT42 under natural infection conditions. Previous studies have reported the use of *Pseudomonas* spp. to control sugarcane smut. Liu et al. [17] isolated *Pseudomonas* strain ST4 from the sugarcane rhizosphere and demonstrated that adding 2% exogenous glucose significantly enhanced its biocontrol efficacy in pot experiments, showing 77% higher disease control compared to treatments without glucose supplementation. In this study, two *Pseudomonas* strains—2143-2 (*P*. *baetica*) and Y8-2 (*P*. *reinekei*)—were evaluated in pot experiments for their biocontrol potential against sugarcane smut. Both strains demonstrated effective disease suppression, with Y8-2 showing outstanding performance, achieving 87.26% control efficacy for cultivar ROC22 and complete protection (100%) for LC05-136. Field inoculation trials revealed 68.40 and 73.99% control efficacy for ROC22 and LC05-136, respectively, and natural infection trials demonstrated 65.73% control for susceptible cultivar KY1. In this study, the biocontrol efficacy of strain 2143-6 observed in the natural field trial at Jinxing Farm (25.09%) was markedly lower than that recorded in the pot experiment. This discrepancy may be attributed to environmental conditions that are less favorable for the reproduction and colonization of the biocontrol agent, as well as to its interactions with the indigenous microbial community. Given the limited dataset obtained from the field trials, these findings should be regarded as preliminary. Further studies, including expanded field evaluations, are required to confirm and refine these results.

Pathogen infection induces physiological changes in host plants, as the activity of antioxidant enzymes and the content of oxidative substances in plant leaves reflect the plant’s disease resistance level [31,32]. Similarly, sugarcane infected with *S*. *scitamineum* undergoes physiological changes [33]. Antioxidant enzyme activities serve as effective physiological indicators for studying smut disease resistance in sugarcane [34]. This study found that in both tested sugarcane cultivars, the leaves of plants inoculated with antagonistic bacteria showed significantly higher SOD and POD activities compared to CK2 (non-bacterial control). Additionally, the H_2_O_2_ content was reduced relative to CK2, indicating that the antagonistic bacteria enhanced the antioxidant enzyme activities in sugarcane leaves, which alleviated excessive reactive oxygen species (ROS) production caused by *S*. *scitamineum* infection and thereby protected the sugarcane plants. MDA, as a product of membrane lipid peroxidation, indicates plant cell membrane damage and is commonly used to assess the extent of plant stress [35]. In the pot biocontrol experiments in this study, the MDA content under CK2 was consistently higher than that under antagonistic bacteria-inoculated treatments, indicating that applying antagonistic bacteria during sugarcane growth actively counteracts *S*. *scitamineum* infection and mitigates cellular damage in sugarcane plants.

Biocontrol bacteria protect plants from diseases and promote plant growth [36,37]. This study found that inoculation with biocontrol strains significantly improved root system characteristics (Figure S2) and enhanced sugarcane leaf photosynthesis (Figures S3 and S4), stimulating sugarcane growth. Many biocontrol bacteria exhibit broad-spectrum activity [38,39]. For instance, three endophytic bacterial strains (*B*. *subtilis*, *Enterococcus faecalis*, and *Paenibacillus paeoniae*) isolated from tomato seeds demonstrate wide-ranging antimicrobial properties. In this study, antifungal assays against five sugarcane fungal pathogens (in addition to *S*. *scitamineum*) revealed that among the five identified biocontrol strains, only Y8-2 showed highly specific inhibition against sugarcane smut. The other four strains, particularly 2143-2, 2143-4, and Y8-3, demonstrated broad-spectrum inhibitory effects against sugarcane smut and the five other tested fungal pathogens.

This study identified effective bacterial strains for the biological control of sugarcane smut, laying the foundation for eco-friendly disease management. In future studies, we will conduct further field trials with these biocontrol strains to fully determine their potential for managing sugarcane diseases. Concurrently, we will investigate their mechanisms of action to provide practical solutions, technical support, and theoretical foundations for the biological control of major sugarcane diseases.

## 5. Conclusions

This study isolated, screened, and identified the following five bacterial strains from sugarcane rhizosphere soil that effectively inhibit the sexual mating and teliospore germination of *S*. *scitamineum*: 2143-2 (*P*. *baetica*), 2143-4 (*B*. *subtilis*), 2143-6 (*B*. *diffusa*), Y8-2 (*P*. *reinekei*), and Y8-3 (*B*. *amyloliquefaciens*). Among them, strains 2143-2, 2143-4, and Y8-3 also exhibited good inhibitory effects against five other major sugarcane diseases. Multi-level control efficacy tests confirmed that the above five bacterial strains exhibited varying degrees of control efficacy against sugarcane smut disease. Among them, strains Y8-2 (*P. reinekei*) and 2143-4 (*B. subtilis*) showed the best control efficacy, demonstrating potential for future application. In addition, inoculation with biocontrol bacteria significantly increased the SOD and POD activities in sugarcane leaves, reduced the H_2_O_2_ and MDA contents, may contribute to enhanced sugarcane disease resistance, and decreased membrane lipid peroxidation damage, thus benefitting sugarcane growth and alleviating the cane yield loss and sugar Brix reduction caused by smut. This study identified several promising biocontrol strains for the management of sugarcane smut, providing a basis for the development of more environmentally sustainable control strategies. Further research, including additional natural field trials, is needed to assess the practical efficacy of these strains under field conditions and to strengthen their potential application in sugarcane smut management.

## Figures and Tables

**Table 1:** Inhibition width and inhibition rates of biocontrol bacteria against pathogenic fungi causing sugarcane diseases.

Strain	*N. sphaerica*		*C. falcatum*		*B. setariae*		*C. ischaemi*		*F. verticillioides*	
	Inhibition Width (mm)	Inhibition Rate (%)	Inhibition Width (mm)	Inhibition Rate (%)	Inhibition Width (mm)	Inhibition Rate (%)	Inhibition Width (mm)	Inhibition Rate (%)	Inhibition Width (mm)	Inhibition Rate (%)
2143-2	16.37 ± 0.40 a	77.16 ± 0.03 a	14.27 ± 0.38 a	72.55 ± 0.02 a	9.17 ± 0.76 a	74.51 ± 0.05 a	11.47 ± 0.50 a	70.98 ± 0.02 a	12.33 ± 0.58 ab	66.27 ± 0.02 b
2143-4	16.33 ± 0.35 a	76.53 ± 0.03 a	14.07 ± 0.31 a	69.41 ± 0.01 a	8.53 ± 0.50 a	72.16 ± 0.06 a	11.80 ± 0.72 a	70.20 ± 0.03 a	11.83 ± 0.76 b	70.59 ± 0.04 ab
2143-6	5.30 ± 0.61 c	53.34 ± 0.06 b	3.63 ± 0.58 b	50.98 ± 0.05 b	5.83 ± 0.76 b	59.22 ± 0.04 b	5.17 ± 1.00 b	59.76 ± 0.07 b	8.40 ± 0.53 c	20.00 ± 0.03 c
Y8-2	0.00 ± 0.00 d	7.25 ± 0.04 c	0.00 ± 0.00 c	7.45 ± 0.01 c	0.00 ± 0.00 c	0.00 ± 0.00 c	0.00 ± 0.00 c	0.00 ± 0.00 c	0.00 ± 0.00 d	0.00 ± 0.00 d
Y8-3	14.57 ± 0.51 b	69.78 ± 0.09 a	13.67 ± 0.58 a	68.24 ± 0.03 a	9.10 ± 0.36 a	70.98 ± 0.09 a	12.33 ± 1.15 a	73.14 ± 0.02 a	13.20 ± 1.06 a	71.37 ± 0.04 a

Note: Different lowercase letters represent a significant difference at the 0.05 level.

**Table 2:** Latent period, disease incidence, and control efficacy of sugarcane smut in the pot experiment.

Cultivar	Treatment	Latent Period (d)	Disease Incidence (%)	Control Efficacy (%)
ROC22	CK1	—	0.00 ± 0.00 c	—
	CK2	106	66.67 ± 8.33 a	0.00 ± 0.00 b
	T1	113	11.11 ± 11.11 ab	83.36 ± 16.64 a
	T2	141	8.33 ± 8.33 ab	87.51 ± 12.49 a
	T3	113	11.11 ± 11.11 ab	83.36 ± 16.64 a
	T4	141	8.33 ± 8.33 ab	87.51 ± 12.49 a
	T5	120	27.78 ± 2.78 ab	58.37 ± 4.15 a
LC05-136	CK1	—	0.00 ± 0.00 c a	—
	CK2	106	66.67 ± 8.33 a	0.00 ± 0.00 c
	T1	141	16.67 ± 8.33 bc	75.01 ± 12.49 b
	T2	—	0.00 ± 0.00 c	100.00 ± 0.00 a
	T3	113	16.67 ± 8.33 bc	75.01 ± 12.49 b
	T4	—	0.00 ± 0.00 c	100.00 ± 0.00 a
	T5	113	27.78 ± 2.78 b	58.37 ± 4.15 b

Note: Different lowercase letters represent a significant difference at the 0.05 level.

**Table 3:** Latent period, disease incidence, and control efficacy of sugarcane smut in the field inoculation experiment.

Cultivar	Treatment	Latent Period (d)	Disease Incidence (%)	Control Efficacy (%)
ROC22	CK1	—	0.00 ± 0.00 d	—
	CK2	105	42.20 ± 1.96 a	0.00 ± 0.00 c
	T2	138	15.83 ± 3.82 bc	62.48 ± 9.05 ab
	T3	109	18.61 ± 1.23 b	55.90 ± 2.92 b
	T4	131	13.33 ± 2.22 c	68.40 ± 5.26 a
LC05-136	CK1	—	0.00 ± 0.00 d	—
	CK2	103	41.92 ± 7.31 a	0.00 ± 0.00 c
	T2	128	12.63 ± 1.59 bc	69.86 ± 3.80 a
	T3	118	16.88 ± 1.21 b	59.73 ± 2.89 b
	T4	133	10.90 ± 1.71 c	73.99 ± 4.09 a

Note: Different lowercase letters represent a significant difference at the 0.05 level.

**Table 5:** Sugarcane Brix and cane yield in the pot experiment.

Cultivar	Treatments	Brix (%)	Cane Yield (g/pot)
ROC22	CK1	17.50 ± 0.31 a	4160.5 ± 51.8 a
	CK2	13.65 ± 0.45 c	2452.8 ± 110.5 c
	T1	15.78 ± 0.31 b	3510.5 ± 271.7 b
	T2	16.58 ± 0.38 ab	3711.6 ± 129.9 ab
	T3	15.90 ± 0.60 b	3659.8 ± 222.8 ab
	T4	16.88 ± 0.59 ab	3730.7 ± 71.8 ab
	T5	16.05 ± 0.41 b	3451.6 ± 121.5 b
LC05-136	CK1	17.35 ± 0.32 a	4231.8 ± 90.1 a
	CK2	13.85 ± 0.22 b	2472.5 ± 120.1 c
	T1	16.78 ± 0.20 a	3572.9 ± 261.7 b
	T2	17.43 ± 0.35 a	4413.9 ± 90.5 a
	T3	17.35 ± 0.17 a	3512.2 ± 161.1 b
	T4	17.35 ± 0.38 a	4040.4 ± 20.9 a
	T5	17.05 ± 0.21 a	3572.8 ± 41.1 b

Note: Different lowercase letters represent a significant difference at the 0.05 level.

## Data Availability

The authors confirm that the data supporting the findings of this study are available within the article and its Supplementary Materials.

## Supplementary Materials

The following supporting information can be downloaded at: https://www.mdpi.com/article/10.3390/plants15132091/s1. Table S1. PCR primers and amplification program; Figure S1. Phylogenetic tree of antagonistic bacterial strains based on 16S rRNA gene sequences; Figure S2. Effects of antagonistic bacteria on root traits of sugarcane cultivars; Figure S3. Effects of biocontrol bacterial strains on photosynthetic parameters of sugarcane cultivar ROC22 during key growth stages; Figure S4. Effects of biocontrol bacterial strains on photosynthetic parameters of sugarcane cultivar LC05-136 during key growth stages.
