# Supplementary material for: Isolationand Identification of Antagonistic Bacteria Against Sporisorium scitamineum and Their Biocontrol Effect on Sugarcane Smut
Source: Plants (Basel). 2026 Jul 5;15(13):2091. doi: 10.3390/plants15132091 (PMC13363828; doi:10.3390/plants15132091)
Supplement: Supplementary file 1 [file plants-15-02091-s001.zip › plants-4354353-supplementary.pdf]

Supplementary materials

Table S1. PCR primers and amplification program

Gene	Primer	Sequence(5'-3')	Product size (bp)	Amplification program
16S rRNA	27F<br>1492R	AGAGTTTGATCCTGGCTCAG<br>TACGGCTACCTTGTTACGACTT	1400-1500	(1) Pre-denaturation: 95℃ for 3 min, (2) Cycling(35 cycles): Denaturation: 95℃ for 15 sec, Annealing: 55℃ for 15 sec, Extension: 72℃ for 90 sec, (3) Final extension: 72℃ for 5 min
	338F<br>806R	ACTCCTACGGGAGGCAGCAG<br>GGACTACHVGGGTWTCTAAT	400-500	(1) Pre-denaturation: 95℃ for 3 min, (2) Cycling(35 cycles): Denaturation: 95℃ for 15 sec, Annealing: 55℃ for 15 sec, Extension: 72℃ for 30 sec, (3) Final extension: 72℃ for 5 min

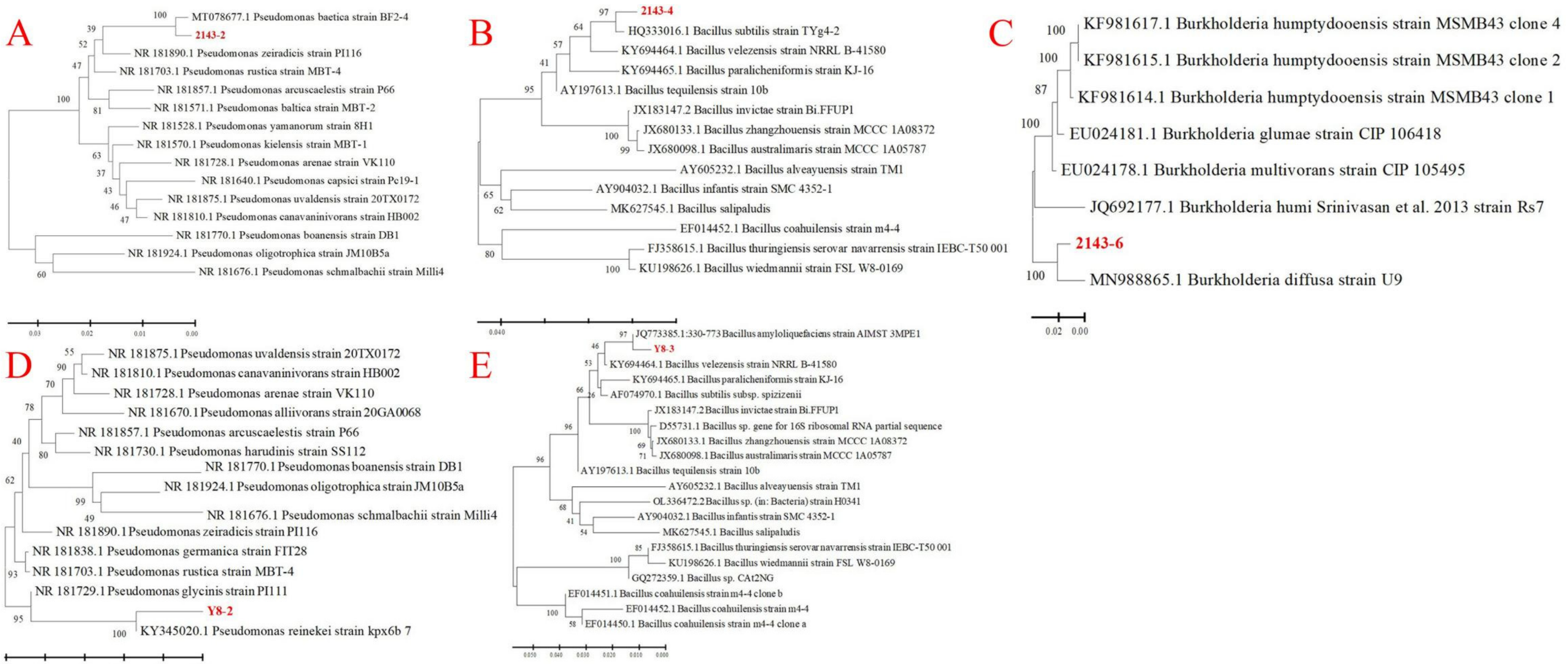

Figure S1. Phylogenetic tree of antagonistic bacterial strains based on 16S rRNA gene sequences.

Note: Strains marked in red represent the bacterial isolates obtained in this study. A. 2143-2; B. 2143-4; C. 2143-6; D. Y8-2; E. Y8-3.

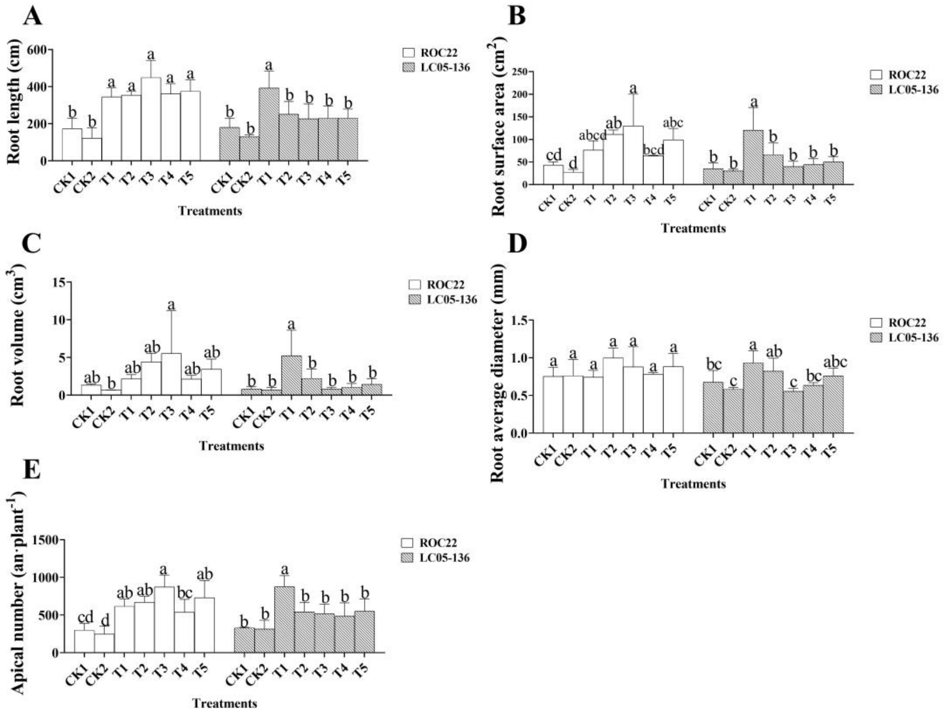

Figure S2. Effects of antagonistic bacteria on root traits of sugarcane cultivars.

Notes: (1) A. Root length; B. Root surface area; C. Root volume; D. Root average diameter; E. Apical number. (2) CK1. Sterile water control; CK2. Teliospore suspension only; T1. CK2 + strain 2143-2; T2. CK2 + strain 2143-4; T3. CK2 + strain 2143-6; T4. CK2 + strain Y8-2; T5. CK2 + strain Y8-3. The bar represents the standard error from three independent replicates in each group, and different lowercase letters represent a significant difference at the 0.05 level.

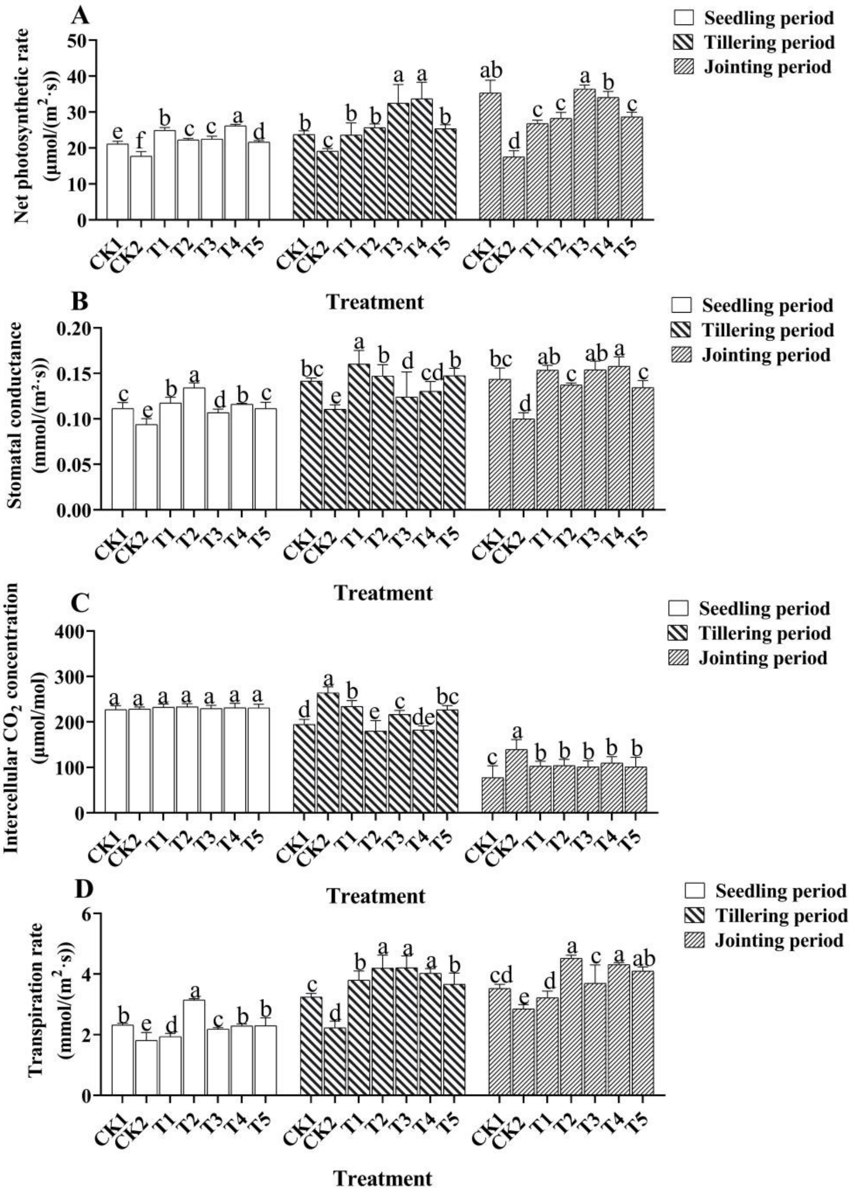

Figure S3. Effects of biocontrol bacterial strains on photosynthetic parameters of sugarcane cultivar ROC22 during key growth stages

Notes: (1) A. Net photosynthetic rate (Pn); B. Stomatal conductance (Gs); C. Intercellular CO_2 concentration (Ci); D. Transpiration rate (Tr). (2) CK1. Sterile water control; CK2. Teliospore suspension only; T1. CK2 + strain 2143-2; T2. CK2 + strain 2143-4; T3. CK2 + strain 2143-6; T4. CK2 + strain Y8-2; T5. CK2 + strain Y8-3. The bar represents the standard error from three independent replicates in each group, and different lowercase letters represent a significant difference at the 0.05 level.

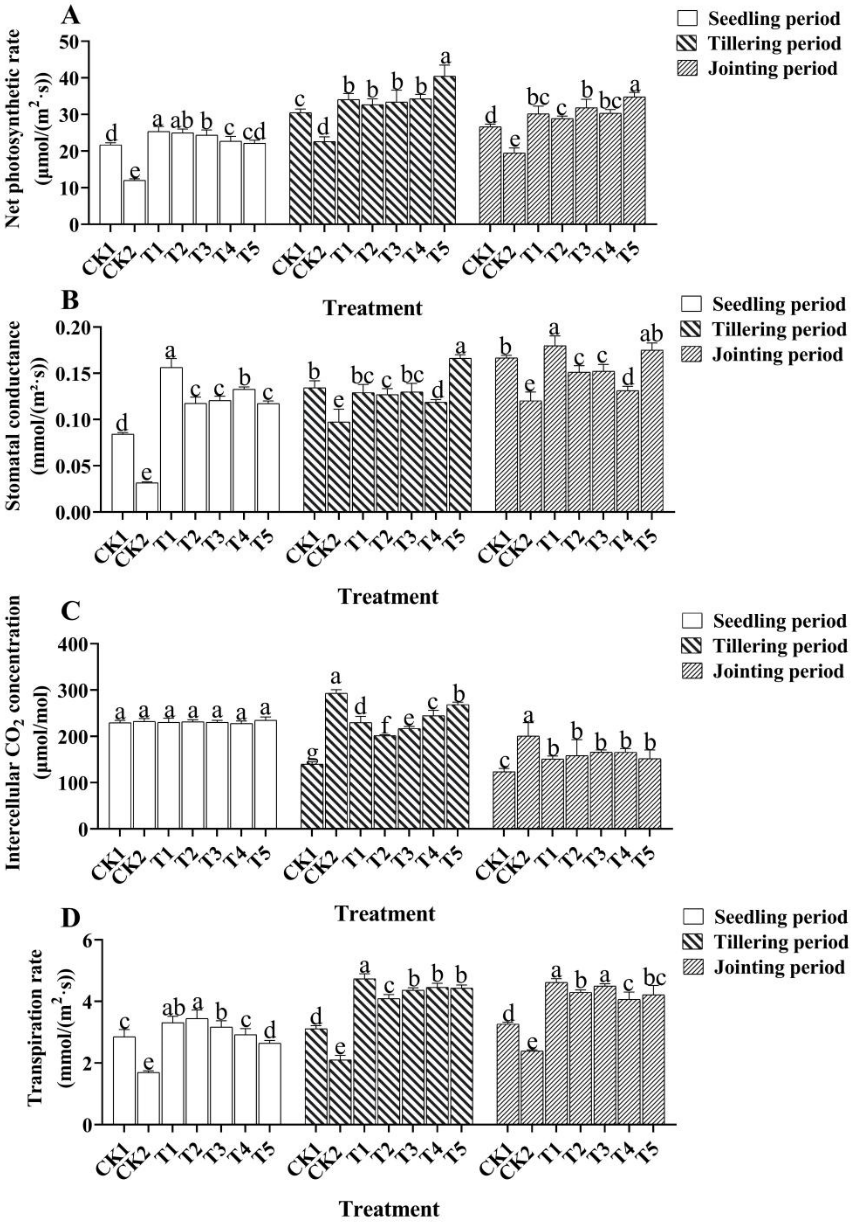

Figure S4. Effects of biocontrol bacterial strains on photosynthetic parameters of sugarcane cultivar LC05-136 during key growth stages

Notes: (1) A. Net photosynthetic rate (Pn); B. Stomatal conductance (Gs); C. Intercellular CO_2 concentration (Ci); D. Transpiration rate (Tr). (2) CK1. Sterile water control; CK2. Teliospore suspension only; T1. CK2 + strain 2143-2; T2. CK2 + strain 2143-4; T3. CK2 + strain 2143-6; T4. CK2 + strain Y8-2; T5. CK2 + strain Y8-3. The bar represents the standard error from three independent replicates in each group, and different lowercase letters represent a significant difference at the 0.05 level.
